# Individual Ranging Behaviour Patterns in Commercial Free-Range Layers as Observed through RFID Tracking

**DOI:** 10.3390/ani7030021

**Published:** 2017-03-09

**Authors:** Hannah Larsen, Greg M. Cronin, Sabine G. Gebhardt-Henrich, Carolynn L. Smith, Paul H. Hemsworth, Jean-Loup Rault

**Affiliations:** 1 Animal Welfare Science Centre, Faculty of Veterinary and Agricultural Sciences, University of Melbourne, Melbourne, VIC 3010, Australia; hlarsen@student.unimelb.edu.au (H.L.); phh@unimelb.edu.au (P.H.H.); 2 School of Life and Environmental Sciences, University of Sydney, Camden, NSW 2570, Australia; greg.cronin@sydney.edu.au; 3 Research Centre for Proper Housing: Poultry and Rabbits, Division of Animal Welfare, Veterinary Public Health Institute, University of Bern, 3052 Zollikofen, Switzerland; sabine.gebhardt@vetsuisse.unibe.ch; 4 Department of Biological Sciences, Macquarie University, Marsfield, NSW 2109, Australia; klynn.smith@mq.edu.au

**Keywords:** free range, radio frequency identification, individual, variation, time budget, pasture, eggs, poultry

## Abstract

**Simple Summary:**

Understanding of how free-range laying hens on commercial farms utilize the outdoor space provided is limited. In order to optimise use of the range, it is important to understand whether hens vary in their ranging behaviour, both between and within individual hens. In our study, we used individual tracking technology to assess how hens in two commercial free-range flocks used the range and whether they varied in their use of the range. We assessed use of three areas at increasing distance from the shed; the veranda [0–2.4 m], close range [2.4–11.4 m], and far range [>11.4 m]. Most hens accessed the range every day (68.6% in Flock A, and 82.2% in Flock B), and most hens that ranged accessed all three areas (73.7% in Flock A, and 84.5% in Flock B). Hens spent half of their time outside in the veranda adjacent to the shed. We found that some hens within the flocks would range consistently (similar duration and frequency) daily, whereas others would range inconsistently. Hens that were more consistent in their ranging behaviour spent more time on the range overall than those that were inconsistent. These different patterns of range use should be taken into account to assess the implications of ranging for laying hens.

**Abstract:**

In this exploratory study, we tracked free-range laying hens on two commercial flocks with Radio Frequency Identification (RFID) technology with the aim to examine individual hen variation in range use. Three distinct outdoor zones were identified at increasing distances from the shed; the veranda [0–2.4 m], close range [2.4–11.4 m], and far range [>11.4 m]. Hens’ movements between these areas were tracked using radio frequency identification technology. Most of the hens in both flocks (68.6% in Flock A, and 82.2% in Flock B) accessed the range every day during the study. Of the hens that accessed the range, most hens accessed all three zones (73.7% in Flock A, and 84.5% in Flock B). Hens spent half of their time outdoors in the veranda area. Within-individual consistency of range use (daily duration and frequency) varied considerably, and hens which were more consistent in their daily range use spent more time on the range overall (*p* < 0.001). Understanding variation within and between individuals in ranging behaviour may help elucidate the implications of ranging for laying hens.

## 1. Introduction

Free-range egg production has increased rapidly over the last decade, being popular with consumers that hold the view that hens are ‘happier and healthier’ in free-range housing systems [1]. The range provides a larger area for hens to roam and interact with or avoid conspecifics, and an environment to express behaviours such as foraging, dust bathing, and sun bathing. Nevertheless, the scientific understanding of how hens in these systems actually use the range is limited. 

The question of how many and how much hens access the range is central to free-range housing systems and has been investigated in a number of recent studies in both experimental and commercial flocks [2,3,4,5,6,7,8,9,10,11,12,13,14,15]. Assessing the use of the range in hens has traditionally been done through point sampling observation methods of the entire flock at one point in time or an average over time. However, this method might not capture the true numbers of hens that access the range overall, given the inability to identify individuals in large flocks, and it may miss crucial information about individual variability in range use. Using these point sampling methods, access to the range in free-range flocks has been reported to be relatively low, generally suggesting that less than 50% of the flock uses the range [4,5,6,7,8]. It has also been widely demonstrated that the majority of hens will remain in the area closest to the shed [5,6,7,8,9]. For example, in a recent survey on six farms in the UK that assessed range usage, 43.2% of the hens observed in the range were within 10 m of the shed, 34.4% were within 10 to 50 m, and 22.4% were further than 50 m from the shed [10]. However, it is not always clear when assessing hen behaviour at flock level whether there are marked differences between individuals in terms of daily behavioural time budgets or whether hens in the same flock have similar patterns of behaviour. Understanding individual differences in ranging behaviour and the factors that affect it is important to elucidate the implications of ranging behaviour for laying hens.

Advances in tracking technology have provided more accurate assessments of how individual laying hens use the range in free-range systems [2]. Radio Frequency Identification Technology (RFID) is currently the most popular method of tracking individual hens and has been implemented in both experimental and commercial flocks [2,3]. In experimental flocks, approximately 80% of the flock has been found to access the pop hole [11], and 50% to 90% of the flock will access the range [12,13,14]. Moreover, in these studies, distinct groups of hens (low and high users of the range) have been identified, indicating that there is marked variation between individual hens in terms of range use. For example, in a study on commercial farms in Switzerland, distinct groups of high and low users were identified, based on overall frequency and duration of visits, even though most hens entered the veranda (also known as a wintergarden) and range at least once [15]. These initial studies using RFID tracking of free-range laying hens indicate that while most hens use the outdoor area, there is considerable variation between individuals within a flock in terms of range use. Individual tracking of hens can also allow for examining patterns of range use, such as time of first access to the range [15] and variation due to time of day and weather [16] on frequency and duration of range access [12,15,17]. Fowl, both domestic and wild, are more active in the morning and evening periods of the day. During these times, foraging is the prominent behaviour, particularly in the evening [18,19,20,21,22], which may be linked with higher range use. 

The aims of this exploratory study were to examine individual variations in ranging behaviour, in terms of both access to the range and the extent of ranging within the flocks, and specifically to describe individual hen movement between three distinct zones within the range; the veranda, close range (immediately adjacent to the veranda), and far range (11.4 m from the shed). Previous research using RFID technology has focused on estimating the proportion of the flock that accessed the range and identifying sub-populations, whereas this is the first study to-date that investigates the use of three distinct range areas and explores the differences in individual ranging behaviour in relation to frequency, duration, and distance using RFID technology. 

## 2. Materials and Methods 

### 2.1. Animals and Husbandry

All methods and animals used in this study were approved by the University of Melbourne Animal Ethics Committee (ethics committee approval number: 1413428.3).

The current study examines access to the range in two free-range laying hen flocks on one commercial farm in southeast Australia (Victoria). Both flocks consisted of about 18,000 Hy-Line Brown laying hens each (referred to as flocks A and B from here on), were considered to be representative of the most common production systems for free-range hens in Australia, and were selected at random based on availability. Each flock had *ad libitum* access to feed and water inside and access to an outdoor area from approximately 1000 h to 1800 h daily (sunrise between 0600 h and 0700 h, sunset between 1700 h and 1800 h), starting from 21 weeks of age. Flock A was 41 weeks of age at the commencement of the study, and hen-day egg production was 93.6%, according to producer records. Flock B was 63 weeks of age, and hen-day egg production was 85.3% at the commencement of the study. Both flocks had access to nest boxes with a single tier slatted floor inside the shed; however, Flock B had access to approximately 1 m of perch space per 47 hens, whereas Flock A did not have access to perches. All hens in both flocks had infra-red laser beak treatment at one day-old.

### 2.2. Study Area

**Figure 1:**
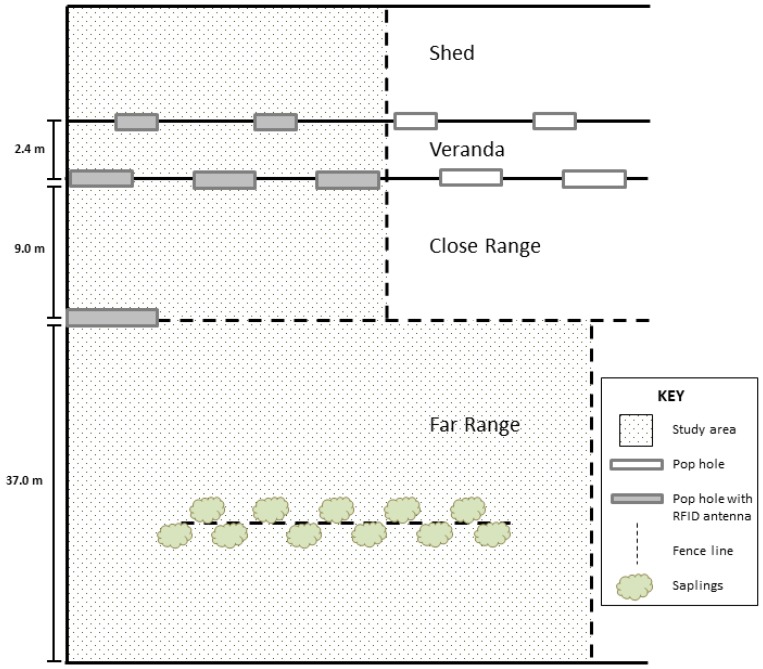
Diagram of the commercial sheds outlining the segregated study area, the three distinct outdoor zones (veranda, close range, and far range), and the location of the pop holes and RFID antennas (not to scale). Both sheds had identical indoor, pop hole, and range dimensions.

Within each flock, a group of 2000 hens was segregated using temporary fencing in both the shed and range, as there was insufficient RFID equipment to cover all exits from the entire shed. The indoor stocking density of the hens in this area was kept consistent with the rest of the shed at approximately 12.1 hens per square metre. Antennas for the RFID system were placed at two pop holes (2 × 0.45 m; Figure 1) giving access from the indoor shed area (5.5 × 30 m) to the veranda area (2.4 × 30 m), at three pop holes (3.65 × 0.45 m) giving access from the veranda area to the ‘close range’ (9 × 30 m), and across a gate (3.65 m wide) giving access from the close range to the ‘far range’ (37 × 41 m). The veranda area was characterised by opaque overhead cover, concrete floors with straw-based litter, and open wire fencing on the close range side. No lighting, temperature control, feed, or water was provided in the veranda area. The close range in both flocks was characterised by bare earth with patches of small rock and gravel areas. The far range in both flocks consisted of a mixed area of bare earth, rocks and gravel, small grasses, and Juncaceae plants with a short fence line through the middle supporting plantings of small Eucalyptus saplings. Hens were segregated from the main Flock And antennas fitted to all pop holes 14 days prior to data collection in Flock A and 11 days prior to data collection in Flock B to allow hens to habituate to the fencing and antennas. 

### 2.3. RFID Antennas

The RFID system used was the Gantner Pigeon System with a bespoke program, Chicken Tracker (© 2015 Gantner Pigeon Systems GmbH, Benzing, Schruns, Austria), which was developed for the use of tracking commercial fowl. This tracking system has previously been validated for tracking individual laying hens and successfully used to track laying hens on commercial farms in Switzerland [15,17]. Antennas (76.5 cm × 30 cm) were attached to both sides of all pop holes and the gate in order to determine the number and the direction of hen movements between zones. Hens were registered as entering (or exiting) an area when they registered on the two opposing antennas in succession. Antennas were made of non-slippery plastic and fixed to the shed slats or ground, forming a small step 2.3 cm high.

### 2.4. Ranging Data Collection

Data were collected for Flock A in August (Australian winter) and one month later from Flock B (Australian spring). The outdoor weather (obtained from weather station 24.7 km from shed) and indoor temperature and lighting conditions experienced by both flocks are presented in Table 1. Laying hens were randomly selected (by visually identifying an individual and then selecting the hen two individuals to the left to avoid observer bias) from one of 10 randomly chosen (via random number generator) locations inside the shed and veranda and then caught with a hand held net. If the first randomly selected hen was not successfully caught, another hen within that location was randomly chosen and caught using the same method. On the day of tagging, hens were temporarily restricted to the indoor shed and veranda to ensure that the hens, which might have otherwise been in the close or far range, could be easily captured and included in the random population sampling. Hens were then fitted with silicone leg bands, each containing a unique ID chip identification number (Ø4.0/34.0 mm Hitag S 2048 bits, 125 kHz) that registered on the antennas as hens walked across them. To retrieve the tags easily at the end of the study, blue or green stock paint was used to temporarily colour the feathers on the back of the tracked hens. Stock paint had been tested in a previous study [23] to ensure that using stock paint would not interfere with hen welfare or behaviour by increasing the incidence of feather pecking. Leg bands containing RFID chips were retrieved from the hens after 19 days to ensure that the hens had not removed the tags during the study period, or left the study area. In Flock A, 441 hens were tagged to track ranging behaviour, and 450 hens were tagged in Flock B. 

At the end of the study, 353 working chips (80%) were retrieved from the hens in Flock A and 309 working chips (69%) were retrieved from Flock B. The lower number of chips retrieved in Flock B was most likely due to human error in attaching the leg bands, rather than any known shed or range areas that would be likely to cause the leg bands to break or be lost. In Flock A, six of the 19 days (32%) of ranging data were excluded from the final analysis due to the system failing and missing data points, and nine of 19 days (47%) were excluded in Flock B, leaving 13 full days of ranging data for Flock A, and 10 full days of ranging data for Flock B. System failures were not related to adverse weather conditions, management practices, or adverse events in the shed (e.g., unexpected power failures), but rather to known failures in writing data to the computer, missing minutes of time from the data set, or days in which the percentage of missing data points (exit to range without return data) exceeded 35% of the daily data set. 

### 2.5. Statistical Analysis

To identify the time of day, frequency, and duration of hen movements between zones, raw data were cleaned and sorted with the SAS™ statistical program (v9.3, SAS Institute Inc., Cary, NC, USA) using two macros modified from Gebhardt-Henrich et al. [15]. The RFID system used has previously been validated in commercial layer flocks and shown to reliably register hens crossing over the antennas at a speed up to 1.5 m/s [17]. Therefore, the captured dataset may have contained missing data for hens travelling in excess of this speed over the antenna. Missing values, characterised as an entry or exit datum point for a hen without a corresponding exit or entry datum point, were excluded from the dataset. All records that indicated a visit to a zone of less than 10 s were excluded from the dataset to eliminate the chance of including false data points created from hens sitting or walking on the pop hole but not entering a designated zone or from hens that changed direction immediately after starting to enter a zone. 

Microsoft Excel™ (2010, Microsoft Corp., Redmond, WA, USA) and MATLAB™ and Statistics Toolbox Release R2016b (The MathWorks Inc., Natick, MA, USA) were used to generate descriptive ranging data including the number of days hens accessed the range, the number of zones hens accessed, the number of hens accessing the range at different times of day, the time spent on range overall per day and per hour, and the frequency of range visits overall and per day. Statistical data analysis was performed using SPSS statistical software™ (v22, IBM Corp, Armonk, NY, USA). To test if the two flocks differed, chi-square goodness of fit tests were performed, comparing the observed proportion of hens in Flock B to the observed proportion of hens in Flock A that never accessed the range, accessed the range at least once (but not every day), accessed the range every day, and that accessed each zone. The coefficient of variation (CV) for individual daily range duration and individual daily frequency of range visits were calculated to give a standardised measure of dispersion of individual hen variability for these ranging variables. Correlations between ranging variables (number of days and zones hens accessed; total and daily duration and frequency of range visits; and individual coefficient of variation for daily ranging) were performed using Pearson correlation analysis or, if data did not meet assumptions of normality or linearity, using Spearman’s rho analysis for nonparametric correlations. Tests for differences in the numbers of hens accessing range per hour of day, overall duration in range per hour of day, time spent in zones, and zone preferences were calculated using one-way ANOVA and corrected for multiple comparisons using the Bonferroni method. 

## 3. Results

### 3.1. Comparisons Between Flocks

There were significant differences between the two flocks, as indicated by chi-square goodness of fit test, for the number of days the hens accessed the range (χ^2^_(2, *n* = 309)_ = 37.67, *p* < 0.001) and the number of zones hens accessed (χ^2^_(3, *n* = 309)_ = 43.78, *p* < 0.001). Therefore, all subsequent analyses are presented separately for both flocks, with no further comparisons made between flocks.

### 3.2. Overall Access to the Range and Time Spent on the Range

The overall time spent on the range (all zones including veranda) over the course of the study varied greatly between individuals within each flock; hens in Flock A spent a mean of 46 ± 1.1 h ranging between a total duration of 34 s and 83 h outside over the 13 days, and hens in Flock B spent a mean of 30 ± 0.7 h ranging between a total duration of 50 min and 57 h outside over the 10 days. Total duration spent on the range and total frequency of range visits were positively correlated (Flock A, Spearman’s rho = 0.74, *n* = 302, *p* < 0.001; Flock B, Spearman’s rho = 0.55, *n* = 300, *p* < 0.001). 

**Figure 2:**
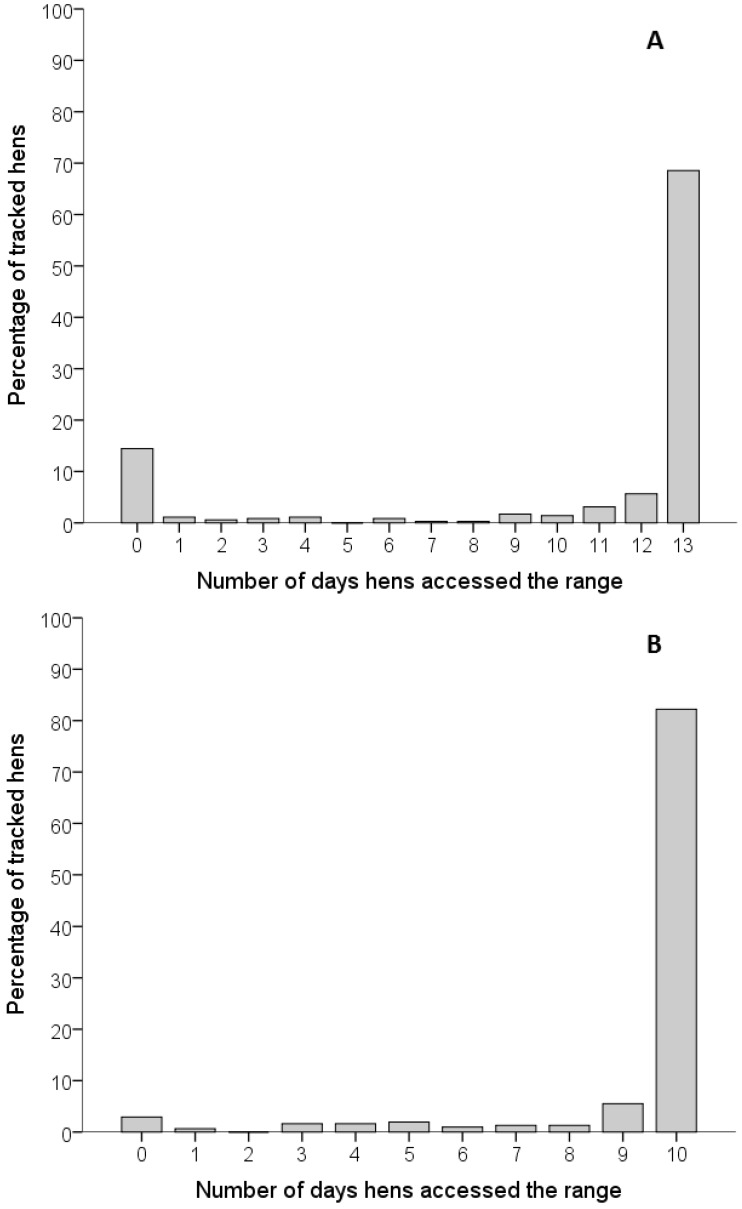
Number of days tracked hens accessed the range. (**A**) Cumulative number of days tracked hens in Flock A entered the range over the course of the 13 day study period; (**B**) Cumulative number of days tracked hens in Flock B entered the range over the course of the 10 day study period.

Most tracked hens in the flocks accessed the range on a regular basis (Figure 2). A total of 68.6% of hens in Flock A and 82.2% of hens in Flock B accessed the range every day during the study (13 and 10 days, respectively), with 80.7% (Flock A) and 91.3% (Flock B) of hens accessing the range on more than half the study days. Only 14.4% and 2.9% of hens never accessed the range over the course of the study in flocks A and B, respectively.

The number of days hens accessed the range was correlated with accessing more outdoor zones (Flock A: Spearman’s rho = 0.46, *n* = 302, *p* < 0.001; Flock B: Spearman’s rho = 0.39, *n* = 300, *p* < 0.001). Additionally, the number of days that hens accessed the range was correlated with both the overall duration of range access (Flock A: Spearman’s rho = 0.62, *n* = 302, *p* < 0.001; Flock B: Spearman’s rho = 0.49, *n* = 300 *p* < 0.001), and the overall frequency of visits to the range (Flock A: Spearman’s rho = 0.64, *n* = 302, *p* < 0.001; Flock B: Spearman’s rho = 0.43, *n* = 300, *p* < 0.001).

### 3.3. Individual Variation in Daily Range Access

**Figure 3:**
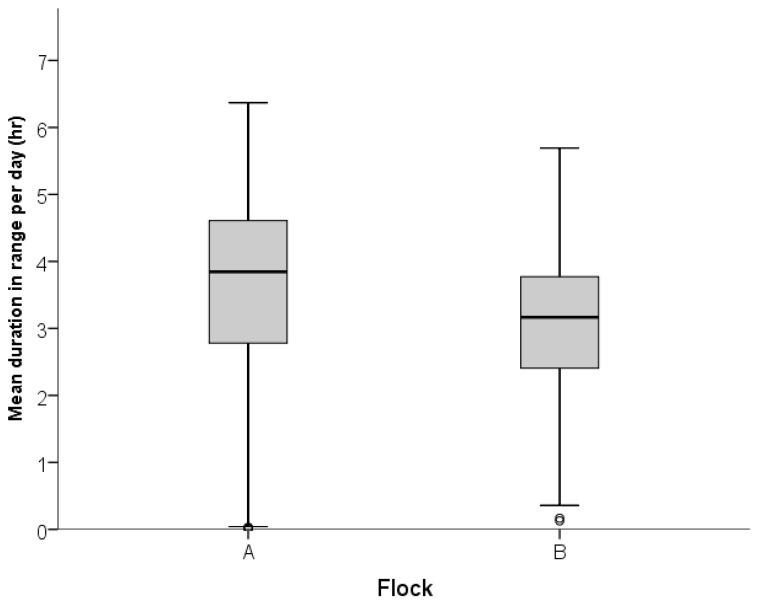
Box plot indicating the distribution of the mean daily duration on the range for individual hens in both flocks. The line in the box indicates the median for each flock, the box indicates the 1st and 3rd quartile, and the whiskers indicate the total range of values. Outliers are indicated using open circles.

Hens differed in terms of the mean time they spent on the range per day (Figure 3), as calculated by total hours spent outside (all zones inclusive) divided by the total number of days an individual hen accessed the range. 

**Figure 4:**
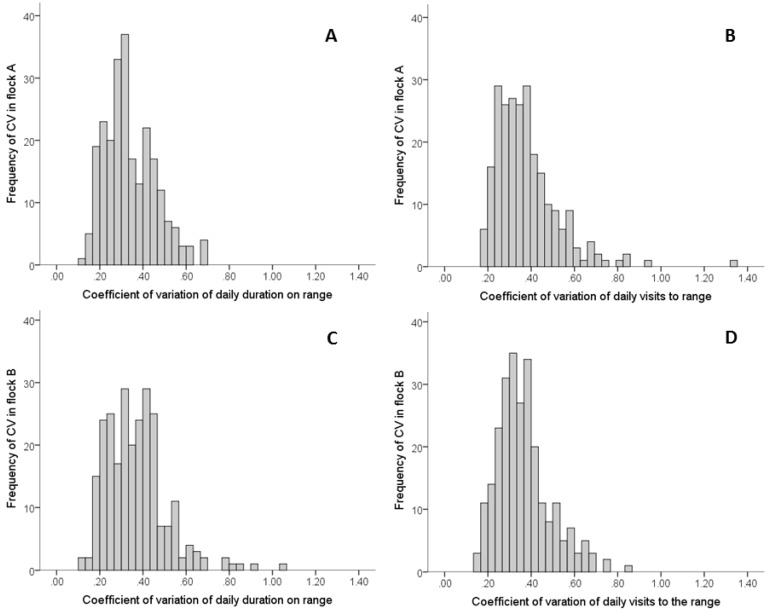
Frequency histograms of coefficient of variation for daily duration and number of visits to the range in Flock A (panels **A** and **B**, respectively) and Flock B (panels **C** and **D**, respectively) for tracked hens that accessed the range every day of the study period (Flock A = 13 days, Flock B = 10 days).

The within-individual hen variability for the daily duration spent on the range, as determined by the coefficient of variation (CV), ranged between 0.10 to 3.61 with a mean of 0.53 ± 0.03 for Flock A and ranged from 0.13 to 3.16 with a mean of 0.49 ± 0.02 for Flock B. The number of days hens accessed the range was negatively correlated with the CV for daily duration (Spearman’s rho = −0.68, *n* = 302, *p* < 0.001 for Flock A and Spearman’s rho = −0.59, *n* = 300, *p* < 0.001 for Flock B). After removing hens that did not access the range every day (*n* = 60 for Flock A and *n* = 46 for Flock B), the CV for daily duration in Flock A ranged between 0.10 to 0.70 with a mean of 0.34 ± 0.01 and in Flock B ranged between 0.13 to 1.04 with a mean of 0.37 ± 0.01 (Figure 4). 

**Figure 5:**
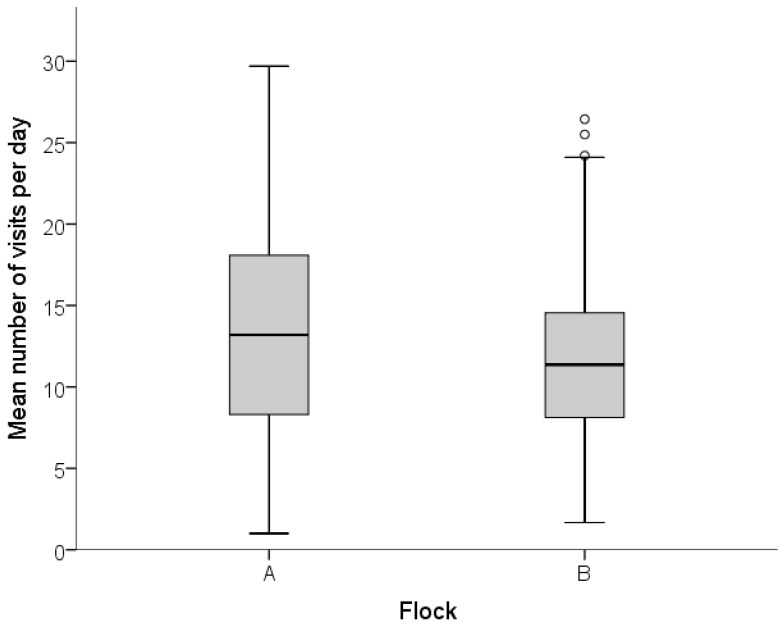
Box plot indicating the distribution of the mean daily frequency of range visits for individual hens in both flocks. The line in the box indicates the median for each flock, the box indicates the 1st and 3rd quartile, and the whiskers indicate the total range of values. Outliers are indicated using open circles.

The mean daily frequency of visits to the range (all zones inclusive) differed between hens (Figure 5).

The within-individual hen variability for daily frequency of visits to the range, as determined by the coefficient of variation, ranged between 0.18 and 3.61 with a mean of 0.55 ± 0.03 for Flock A and ranged from 0.15 and 3.16 with a mean of 0.48 ± 0.02 for Flock B. The coefficient of variation for daily frequency of visits to the range was negatively correlated with the number of days hens accessed the range (Spearman’s rho = −0.65, *n* = 302, *p* < 0.001 for Flock A and Spearman’s rho = −0.58, *n* = 300, *p* < 0.001 for Flock B). After removing hens that did not access the range every day (*n* = 60 for Flock A and *n* = 46 for Flock B) the CV for daily frequency in Flock A ranged between 0.10 to 1.34 with a mean of 0.38 ± 0.01 and in Flock B ranged between 0.15 to 0.84 with a mean of 0.37 ± 0.01 (Figure 4).

**Figure 6:**
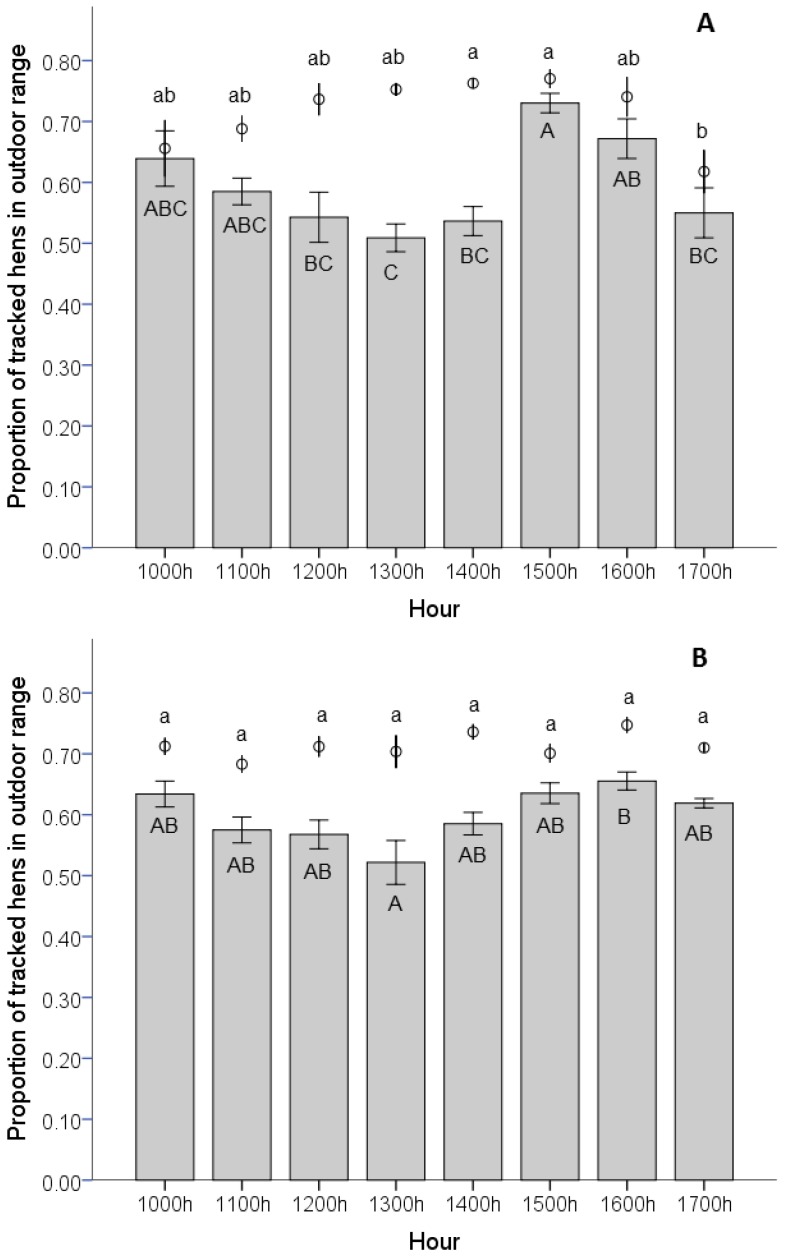
Mean proportion of hens that were outside each hour (1000–1700 h) for Flock A (**A**) and Flock B (**B**). Bars indicate the mean proportion (±SE) of the tracked hens that entered the range during that hour (rate of entry to range). Open circles (φ) indicate the mean proportion (±SE) of the tracked hens that were outside at least once during that hour but did not necessarily enter the range during that hour. Bars with different letters (A–C) indicate significant differences in the rate of entry to the range between those hours (*p* < 0.05), and open circles with different letters (a–b) indicate significant differences in the number of hens outside between those hours (*p* < 0.05).

The number of tracked hens on the range was influenced by the time of day (F(_7,96)_ = 4.1, *p* = 0.001) in Flock A. Significantly fewer tracked hens were on the range during the hour from 1700 h to 1800 h (62.0%), compared with 1400 h to 1500 h (76.3%) and 1500 h to 1600 h (77.0%; *p* < 0.05, Figure 6). The number of tracked hens on the range in Flock B was not influenced by the time of day (F_(7,72)_ = 1.6, *p* = 0.15).

The time of day influenced the proportion of tracked hens that entered the range (rate of entry) in both Flock A (F_(7,96)_ = 5.7, *p* < 0.001) and Flock B (F_(7,72)_ = 4.3, *p* < 0.001; Figure 6). In Flock A, more tracked hens entered the range during the hour from 1500 h to 1600 h (73.0%) than in the hours from 1200 h to 1500 h (<55.0%; *p* < 0.05) and from 1700 h to 1800 h (55.0%, *p* < 0.05). More hens also entered the range from 1600 h to 1700 h (67.2%) than from 1300 h to 1400 h (50.9%; *p* < 0.05). In Flock B, the proportion of tracked hens entering the range differed only between the hours from 1300 h to 1400 h (52.2%) and 1600 h to 1700 h (65.5%; *p* < 0.05). 

The mean duration spent on the range per hour was also influenced by time of day (F_(7,96)_ = 18.5, *p* < 0.001 for Flock A, and F_(7,72)_ = 66.8, *p* ≤ 0.001 for Flock B). Hens in Flock A spent significantly less time on the range from 1500 h to 1600 h (28.3 min) and 1700 h to 1800 h (29.1 min) than at all other times of the day (*p* < 0.05), except for 1000 h to 1100 h (31.1 min). At other times of the day, hens spent between 35.6 and 45.0 min on the range, with the peak occurring from 1300 h to 1400 h. In Flock B hens spent significantly less time from 1500 h to 1600 h (20.1 min) and significantly more time from 1300 h to 1400 h (37.9 min) on the range than at any other times of the day (between 27.9 and 33.2 min; *p* < 0.05). 

The time of day for the first visit to the range was negatively correlated with mean daily duration spent on the range (Flock A: Spearman’s rho = −0.41, *n* = 3676, *p* < 0.001; Flock B: Spearman’s rho = −0.31, *n* = 2838, *p* <0.001) and mean daily frequency of visits (Flock A: Spearman’s rho = −0.51, *n* = 3676, *p* < 0.001; Flock B: Spearman’s rho = 0.39, *n* = 2838, *p* < 0.001). 

### 3.4. Access to the Three Distinct Zones

Most hens in both flocks accessed all three zones monitored in the outdoor range, with significantly fewer hens accessing just the veranda compared to those accessing the veranda and close range in both flocks (Table 2).

Overall, hens in Flock A made significantly longer visits to the veranda than to the far range, and the shortest visits occurred in the close range (*p* < 0.001) (Table 3). Hens in Flock B made significantly longer visits to the far range than to the close range and the veranda (*p* < 0.001) (Table 3). When comparing relative time spent in the various zones of the range as a proportion of total time the flock spent on the range, both flocks spent approximately half the time in the veranda, less time in the close range, and the least proportion of time in the far range (Table 3).

Individual hens also varied in terms of the zones where they spent most time outdoors (zone preference) compared to the flock mean. Of the 260 hens in Flock A that accessed all three zones in the range, 80.8% spent most time outdoors in the veranda than the two other zones, 13.5% of the hens spent most time in the close range, and 5.8% of hens spent most time in the far range. Similarly, in Flock B, of the 261 hens that accessed all three zones, 62.5% spent most time in the veranda, 22.2% spent most time in the close range, and 15.3% spent most time in the far range. 

The overall time hens spent on the range differed between hens with different zone preferences (Flock A; F_(3, 298)_ = 31.4, *p* < 0.001 and Flock B; F_(3, 298)_ = 20.7, *p* < 0.001). Hens in Flock A that spent most time outdoors in the veranda spent significantly less time outside overall (47.1 ± 1.16 h) when compared to hens that spent most time outdoors in the close range (58.0 ± 2.5 h, *p* < 0.01) and those that spent most time outdoors in the far range (58.9 ± 3.1 h, *p* = 0.07). Hens in Flock B that spent most time outdoors in the veranda spent significantly less time in the range overall (30.0 ± 0.8 h) than hens that spent most time outdoors in the far range (35.5 ± 1.5 h, *p* = 0.01) but not less than those that spent most time outdoors in the close range (33.3 ± 1.3 h, *p* = 0.22).

## 4. Discussion

Our study showed little variation between RFID-tracked hens in the number of days they accessed the range, with over 60% of hens in both flocks accessing the range on all available days. However, individual hens varied greatly in the duration of their visit to the range, with some hens in both flocks spending only a few minutes on the range every day, while others spent around six hours on the range daily, and some hens visiting the range once daily, whereas others visited it over 25 times daily. 

In comparison to hens that consistently accessed the range every day, hens that did not access the range every day showed more individual variation in daily ranging patterns. Furthermore, even when considering only the hens that accessed the range every day, there was still considerable variation in the consistency of individual hen ranging patterns. This study is the first to assess within-individual variation in ranging patterns on commercial free-range laying hens. Considering individual consistency in ranging behaviour may be useful to examine the welfare implications of ranging or to predict range use. 

This study incorporated the use of RFID technology on two commercial flocks to track individual hens to determine how they use the range. This technology provides several advantages over more traditional flock estimates of range use because it allows the investigation of more differences between individuals within a flock in terms of ranging patterns. In both flocks in this study, over 60% of the hens accessed the range every day, and 85.6% and 97.1% (Flock A and Flock B, respectively) accessed the range area at least once, which is in contrast to previous research using point sampling that reported that very few hens accessed the range [6,10,20,25,26]. The present results are similar to previous research using individual tracking of hens in both experimental and commercial conditions. While monitoring pop hole use in experimental flocks, 80% of the hens were found to access the pop holes on at least 50% of the days [11], whereas when tracking individual hens in experimental flocks similar to our study, up to 85% of hens were found to access the range on most days [12], and between 66.5% and 80.5% of hens accessed it daily [14]. In commercial Swiss flocks, 79%–99% of the tracked hens were found to access the veranda at least once, and 47%–90% of tracked hens accessed the range beyond the veranda at least once [15]. 

As hens were tracked continuously throughout the study, we were able to examine how the time of day influenced ranging patterns in individual hens. Gebhardt-Henrich et al. [15] found that the sooner hens accessed the range after the pop holes opened, the longer they spent on the range overall, which is supported by our results. We found little difference in the number of hens that accessed the range throughout the day, based on hourly counts; however, the proportion of the hens entering the range declined during the middle of the day (around 1300 h) and late afternoon (around 1700 h) for both flocks and was highest immediately after the pop holes opened (around 1000 h) and during mid-afternoon (around 1500 h). Conversely, the mean duration that hens spent on the range (in total, not per visit), was higher around late morning-midday and lowest in the mid-afternoon. This suggests that hens that go outside mid-morning stay out for longer single visits, whereas hens that enter the range in the midday period made shorter visits to the range. This daily pattern of entering the range is similar to that reported by other studies that indicate that hens are more active in the evening period, a peak time for foraging to occur, than in the midday period [5,19,27,28] but is not consistent with other studies that indicate that fewer hens are seen on the range during the midday period compared to the morning and afternoon [4]. One potential explanation for this disparity is that our numbers include hens that access the veranda as well as other areas of the range, which contains overhead cover and is a highly preferred area [29], potentially attracting more hens to the range. 

The veranda area is a hybrid space with both indoor and outdoor qualities, providing some shelter from the elements while maintaining an outdoor climate [30], allowing hens to perform natural behaviours that cannot be performed indoors (sun bathing, dust bathing, and foraging). Thus, there are valid reasons for considering this space as either part of the indoor enclosure or outdoor area, but studies on the biological relevance of a veranda for hens are lacking. We chose here to classify the veranda as part of the outdoor area because it was managed by the farm as an outdoor area, and therefore hens had the same exposure to the veranda as they did to the rest of the range. Further research on the veranda area in various climates and under different conditions will provide better perspective on how such an area influences hen behaviour, welfare, and flock management.

This study is the first to use RFID technology to track the use of three distinct zones in the range (veranda, close range, and far range) by individual hens, thereby improving our understanding of how hens use various areas of the range. Our results showed that most hens that went outside entered all three zones, travelling at least 11 m from the indoor shed into the far range zone. There was also a moderate correlation between the number of days on which hens accessed the range and the total number of zones hens accessed on the range; hence the more time hens spent on the range, the more likely they were to venture farther from the shed. Previous research found that, at farther distances from the shed, fewer hens could be seen in the range [5,10]. However, the far range in the present study was closer to the shed than in other studies [5,10]; therefore it is possible that fewer hens do access the most distant parts of larger ranges. The flock sampling method used in previous research may also have underestimated the number of hens using areas farther from the shed. Our results indicate that hens spend more time in the veranda, and significantly less time in the far range. Thus, frequent but short visits to the far range reduces the likelihood of recording hens in areas farther from the shed using point sampling techniques. The uneven use of outdoor zones in the present study, in addition to distance from the shed, may also be due to less overall cover in the far and close range. The veranda provides an area with considerable overhead cover close to the shed whilst still remaining outdoors and providing hens in these flocks with areas for dust-bathing, foraging, and sun-bathing. However, the close range and far range in this study were characterised by no overhead cover (except some small saplings in the far range) and predominately gravel/dirt ground cover with sparse areas of hardy grasses. 

Although the largest proportion of time outside was spent in the veranda zone, the mean length of visits to each of the zones differed between the two flocks. In Flock A, the longest visit duration was seen in the veranda, whereas, in Flock B, it was in the far range. There were no discernible differences between the ranges of the two flocks that could explain this. This is also reflected in the proportion of individuals within the flocks that preferred each zone. Far ranging hens were more prevalent in Flock B than Flock A, suggesting that there are other aspects of individual variation and range design that are important for range use that were not examined in this study, i.e., personality (fearfulness, boldness), age, social structure or early experience [4], welfare status, or other factors [3]. Hens in both study flocks had the same management and rearing conditions; however, rearing occurred during different seasons (20 weeks apart), which would have impacted early experience, particularly the season at which hens had first access to the range, and potentially influenced other factors. 

Our study only examined ranging behaviour in hens during two seasons (winter and spring) but was not able to follow hens across multiple seasons. The literature suggests that fewer laying hens access the range during spring [5,22]. However, given the relatively limited understanding of the influence on seasons of hen ranging behaviour, particularly in Australia, we are unable to predict how hens may have accessed the range during different seasons. 

Age was the most identifiable difference between the two flocks in this study; however, it is not possible to identify what impact this may have had on ranging behaviour. Richards et al. [11] found a slight trend towards older hens (55 and 65 weeks of age) accessing the range more than younger hens (35 and 45 weeks of age); however this trend also corresponded with warmer temperatures. Age also had no impact on use of space in individually tracked hens that were followed over their entire lay cycle, but early experience did impact how hens used the range [4,22]. Other factors that may influence ranging behaviour are egg production, changes in diet or dietary needs, changes in management practices, or even stressor events such as heat waves/cold snaps, disease, or predator attacks. Therefore, it is not feasible to identify age or other factors that differ between the two flocks to explain ranging patterns in this study. 

It is unlikely that the RFID tracking system altered the hens’ ranging behaviour during the study, as others have examined the effect of the system on ranging behaviour and found no significant changes with or without the system [12,17]. We cannot rule out that installing temporary fences to form an ‘experimental pen’ and thereby reducing the size of the flock changed the ranging behaviour of the hens. Smaller flock sizes increase the proportion of hens seen in the range [5,22,31,32], as well as increasing the duration spent on the range [15]. However, as we assessed ranging behaviour well after hens had been given access to the range (20 and 40 weeks prior to the study), management (stocking density, pop hole opening times) remained consistent, and our segregated birds had continuous visual contact with the rest of the flock, we do not expect segregation to have affected hens’ ranging behaviour.

The current study is a descriptive study of two laying hen flocks in Victoria, Australia, and thus has limitations for broader interpretations and applications of the results, due to the restricted range of variables examined. However, this paper adds to the current body of work that investigates how laying hens use the range in free-range production systems using individual tracking technology and examines some novel questions such as zone access and individual variation. Understanding ranging behaviour in laying hens has broad implications, such as for hen welfare, but also environmental sustainability, hen health, and food safety. There have been few reported studies that examine the impact of individual range use on welfare characteristics [4,12,33], and currently the results obtained do not demonstrate clear and consistent relationships. Given the noticeable variation between individual hens within a flock that the present results have shown, taking into account variation between individual hens in their range may clarify the implications of ranging for laying hens. 

## 5. Conclusions 

A majority of laying hens accessed the range on a regular basis. However, individual hens within a flock vary considerably in their ranging behaviour patterns in terms of duration and frequency of range visits but less so in the number of days and zones accessed. Hens that were more consistent in their daily range use spent more time outside and made more visits to the range overall compared to hens that varied more in their day-to-day range use. Additionally, most hens in the Flock Accessed all three zones, venturing further than 11 m from the shed, but the majority of hens spent most of their time outside in the veranda adjacent to the shed. Understanding how variation in ranging behaviour within and between individuals correlates with hen welfare and management practices is an important scope for future research. 

## Figures and Tables

**Table 1:** Mean daily weather and photoperiod conditions for the flocks during the study period (13 days of data for Flock A; 10 days of data for Flock B). Indoor parameters were collected from temperature and light monitoring equipment with sensors inside the shed. External weather parameters were obtained from the Australian Government Bureau of Meteorology (BOM) [24].

Weather Variables	Flock A	Flock B
*Outdoor maximum daily ambient temperature (°C)*	11.2 ± 0.6	17.3 ± 0.4
*Outdoor minimum daily ambient temperature (°C)*	2.1 ± 0.9	1.7 ±1.1
*Indoor maximum daily ambient temperature (°C)*	18.1 ± 0.3	21.1 ± 0.5
*Indoor minimum daily ambient temperature (°C)*	13.1 ± 0.4	16.3 ± 0.5
*Daily rainfall (mm) (# days rain registered)*	1.2 ± 0.6 (9)	0 (0)
*Outdoor relative humidity at 0900 h (%)*	85.2 ± 3.7	72.6 ± 3.8
*Outdoor wind speed at 0900 h (km/h)*	4.3 ± 1.0	6.4 ± 1.8
*Indoor light intensity (lux)*	94.7 ± 4.1	83.5 ± 1.4

**Table 2:** Percentage of hens in both flocks that accessed the different zones of the range over the course of the study (13 days for Flock A, 10 days for Flock B). Different superscripts within each flock (row); ^a–c^ indicate a significant difference (*p* < 0.05) in the proportion of hens within the flock that accessed each zone.

	Zones Accessed				
**Percentage of Hens**	*None*	*Veranda Only*	*Veranda + Close Range*	*All Three Zones*	χ^2^
**Flock A (%)**	14.4 ^a^	2.8 ^b^	9.1 ^a^	73.7 ^c^	(*n* = 353) 245.7
**Flock B (%)**	2.9 ^a^	0.3 ^a^	12.3 ^b^	84.5 ^c^	(*n* = 309) 43.8

**Table 3:** Mean duration in the different range zones and proportion of the overall time outdoors spent in each zone; within flocks, values with different superscripts (^a–c^) differ significantly (*p* < 0.05).

	**Zones**			**F-Value**
	*Veranda*	*Close Range*	*Far Range*	
**Flock A**				
*Mean duration of visit to zone (min ± SE)*	19.43 ± 0.20 ^a^	11.85 ± 0.15 ^b^	16.18 ± 0.12 ^c^	433.3
*% time outside in each zone*	57.75	25.85	16.41	
**Flock B**				
*Mean duration of visit to zone (min ± SE)*	13.23 ± 0.17 ^a^	17.70 ± 0.24 ^b^	25.24 ± 0.13 ^c^	476.6
*% time outside in each zone*	47.40	32.09	20.50	
